# Induction of micronuclei by four cytostatic compounds in human hematopoietic stem cells and human lymphoblastoid TK6 cells

**DOI:** 10.1038/s41598-018-21680-8

**Published:** 2018-02-20

**Authors:** Henning Hintzsche, Gracia Montag, Helga Stopper

**Affiliations:** 1 Institute of Pharmacology and Toxicology, University of Wuerzburg, Versbacher Str. 9, 97078 Wuerzburg, Germany; 2 Present Address: Bavarian Health and Food Safety Authority, Eggenreuther Weg 43, 91058 Erlangen, Germany

## Abstract

For mutagenicity testing, primary lymphocytes or mammalian cell lines are employed. However, the true target for carcinogenic action of mutagenic chemicals may be stem cells. Since hematopoietic cancers induced by chemical agents originate at the hematopoietic stem cell (HSC) stage and since one of the side effects of chemotherapeutic cancer treatment is the induction of secondary tumors, often leukemias, HSC may be a suitable cell system. We compared the sensitivity of HSC with the genotoxicity testing cell line TK6 for chromosomal mutations. HSC were less sensitive than TK6 cells for the genotoxic effects of the model genotoxins and chemotherapeutic agents doxorubicin, vinblastine, methyl methanesulfonate (MMS) and equally sensitive for mitomycin C (MMC). However, loss of viability after mitomycin C treatment was higher in HSC than in TK6 cells. Among the factors that may influence sensitivity for genomic damage, the generation or response to reactive oxygen species (ROS) and the effectiveness of DNA damage response can be discussed. Here we show that HSC can be used in a standard micronucleus test protocol for chromosomal mutations and that their sensitivity was not higher than that of a classical testing cell line.

## Introduction

Genotoxicity testing aims at identifying a mutagenic and therefore potentially carcinogenic activity of a substance. Human primary lymphocytes or mammalian cell lines are mainly used for this purpose. Permanent cell lines are either derived from a tumor or have gained the ability to grow for indefinite times because of spontaneous or induced mutations. Therefore, their abilities to regulate the cell cycle, proliferation, and sensitivity for cell death are usually altered. Peripheral lymphocytes on the other hand are differentiated cells. However, the original cells from which chemically induced tumors are developed are most likely stem cells (e.g., White and Lowry^1^, Sell *et al*.^2^). Concerning DNA damage, it is thought that stem cells have very rigid controls and that unrepaired lesions will likely lead to cell death, in order to protect the organism and to keep the stem cell pool genetically intact^3,4^. Because it is highly relevant whether the tested substance is able to induce mutations in stem cells, the question arises whether lymphocytes and cell lines are a suitable surrogate system for stem cells in genotoxicity testing. Sensitivity for the formation and efficiency of repair of chemically induced DNA damage, and sensitivity for entering cell death pathways upon chemical insult may vary between stem cells and cell lines or lymphocytes.

Hematopoietic cancers induced by chemical agents are thought to mostly originate at the hematopoietic stem cell (HSC) stage^5^. About 25 of the 100 known human carcinogens induce leukemias or lymphomas^6^. In addition, one of the side effects of chemotherapeutic cancer treatment is the induction of secondary tumors^7,8^, about 5% of which are leukemias and lymphomas^8^. Therefore, for both aspects, i.e. testing of substances for admission, and assessment of the mutagenicity of chemotherapeutic agents, HSC may be a suitable cell system. However, HSC have not been used extensively for either of these purposes. Here, we compared the sensitivity of a typical mammalian genotoxicity testing cell line, human B lymphoblastoid TK6 cells, with that of HSC. We used micronucleus formation as end point for genotoxicity, because in this test a chromosomal mutation is detected which has already been transmitted to a daughter cell^9,10^ and because the basal micronucleus frequency in peripheral human lymphocytes correlates with the cancer risk on a population level^11^. Micronuclei are chromatin-containing bodies and may contain a fragment of a chromosome or whole chromosome(s). They are the result of DNA damage and may not reflect a chromosome aberration per se. Although the fate of micronucleus containing cells is not known for every situation (i.e. every cell type, inducing chemical…), it is hypothesized that formation of micronuclei can itself be a step in the process of cell transformation^9,10,12^. At least, cells still must be able to proceed through one mitosis after formation of the genomic damage. This is a difference to the analysis of chromosomal aberrations, where cells are analyzed in mitosis and it is not known whether the affected cells would be viable to survive through that mitosis. This is also different from the comet assay, another widely used genotoxicity test^13,14^, in which repairable lesions are detected and it is not always certain that the observed damage is transformed into heritable genomic mutations.

HSCs have been used as toxicological test system^15^, but here we demonstrate the utility of a standard testing micronucleus protocol with chemotherapeutic agents in HSC. One recent publication shows micronucleus data with HSC for radiation exposure^16^, and another paper^17^ had used HSC for micronucleus induction after treatment with 1,4-benzoquinone as a model for benzene-induced hematotoxicity and leukemogenesis. As test compounds, four substances that are being used for chemotherapeutic treatment of cancer (doxorubicin, vinblastine, mitomycin C) or can serve as a model for the class of alkylating agents that are also being used in tumor chemotherapy (methyl methanesulfonate) were applied. We chose compounds with different and well-known, relevant mechanisms of mutagenic activity. Doxorubicin is a mutagenic intercalating and radical-generating anthracycline, the alkaloid vinblastine is a spindle poison and aneugenic agent, MMS is an alkylating agent which is often used as positive control in mutation assays, and the cytotoxic antibiotic MMC is an effective DNA-crosslinker.

## Results

**Figure 1:**
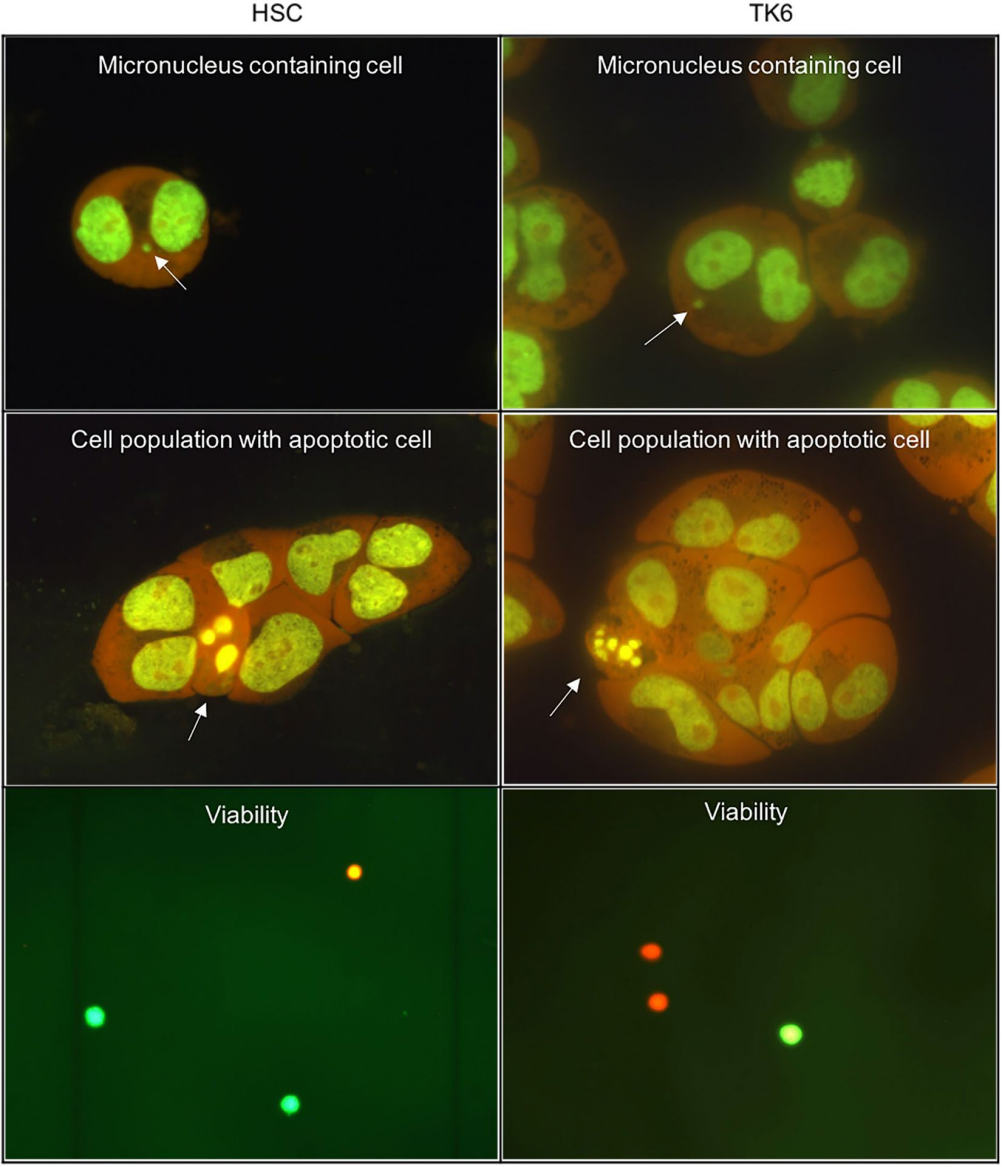
Typical appearance of human hematopoietic stem cells (HSC, left column) and human lymphoblastoid cells (TK6, right column) in the endpoints analyzed here. Shown are binucleated cells containing a micronucleus (upper panel), a cell population with an apoptotic cell (middle panel) and appearance of cells in the viability test (green = viable; red = non viable). Viabilty is assessed at a microscopic magnification of 200-fold, while the other endpoints are assessed at 400-fold. Images are further enlarged for optimal visibility.

In human hematopoietic stem cells (HSC) micronucleated cells, cell proliferation, apoptotic cells and cell viability show a microscopically detectable appearance similar to that seen in the established genotoxicity testing human lymphoblastoid cell line TK6 (Fig. 1).

**Figure 2:**
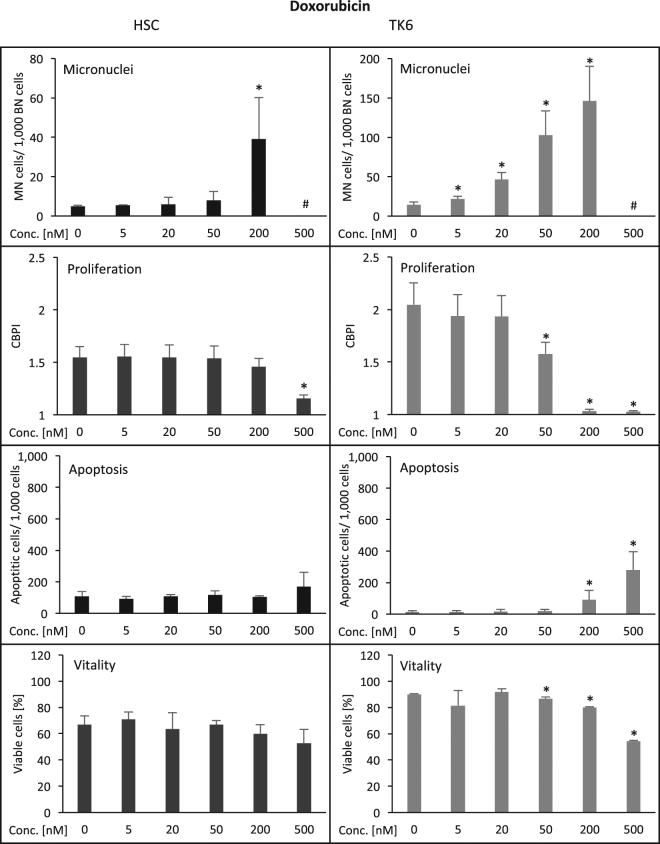
Induction of damage in human hematopoietic stem cells (HSC, left column) and human lymphoblastoid cells (TK6, right column) by doxorubicin. Displayed are the frequency of micronucleated cells/1,000 binucleated cells (MN-cells/1,000 BN-cells), the reduction of cell proliferation (CBPI = cytochalasin B proliferation index), the induction of apoptosis (determined microscopically according to nuclear morphology; number/1,000 cells) and the reduction of cell viability (cell membrane integrity/enzymatic dye activation assay (%). Numbers are averages from three independent experiments with standard deviation. *p ≤ 0.05 (Mann-Whitney-U-Test). ^#^Due to toxicity, insufficient binucleated cells for micronucleus evaluation were available. Y-axes are equal for HSC and TK6 for all endpoints except for micronuclei, where scalings were chosen according to optimal visibility of effects.

The anthracycline doxorubicin induced micronuclei in HSC cells at 200 nM (Fig. 2; Table 1), while TK6 cells exhibited elevated frequencies at 5 nM and higher. Cell proliferation was reduced after treatment with 500 nM doxorubicin in HSC and 50 nM in TK6 cells. Apoptosis induction showed a non-significant trend for elevation in HSC with 500 nM doxorubicin and an increase in TK6 cells with 200 nM doxorubicin. Viability showed a non-significant trend for reduction at 500 nM in HSC and in TK6 cells with 50 nM and higher. Overall, HSC were less sensitive for cellular damage induction by doxorubicin by a factor of 40 for micronucleated cells and 10 for proliferation (Table 1) among the applied doses.

**Table 1:** Lowest effective (p ≤ 0.05) tested doses of chemicals in human hematopoietic stem cells (HSC) and human lymphoblastoid TK6 cells.

	HSC	TK6	Factor dose HSC/dose TK6
**Doxorubicin (nM)**			
Mn-cells/1,000 bn-cells	200	5	40
Proliferation (CBPI)	500	50	10
Apoptosis (o/^oo^)	(500)	200	—
Viability (% viable)	(500)	50	—
**Vinblastine (nM)**			
Mn-cells/1,000 bn-cells	100	10	10
Proliferation (CBPI)	150	20	7.5
Apoptosis (o/^oo^)	200	10	20
Viability (% viable)	(200)	10	—
**MMS (µM)**			
Mn-cells/1,000 bn-cells	250	100	2.5
Proliferation (CBPI)	375	250	1.5
Apoptosis (o/^oo^)	375	100	3.75
Viability (% viable)	375	250	1.5
**MMC (µM)**			
Mn-cells/1,000 bn-cells	0.1	0.1	1
Proliferation (CBPI)	0.5	0.5	1
Apoptosis (o/^oo^)	1	1	1
Viability (% viable)	1	10	0.1

If no significant effect was induced at the tested doses, the maximal tested dose is given, but no dose factor is calculated. Mn-cells/1,000 bn-cells = micronucleated cells/1,000 binucleated cells. MMS = methyl methanesulfonate; MMC = mitomycin C.

**Figure 3:**
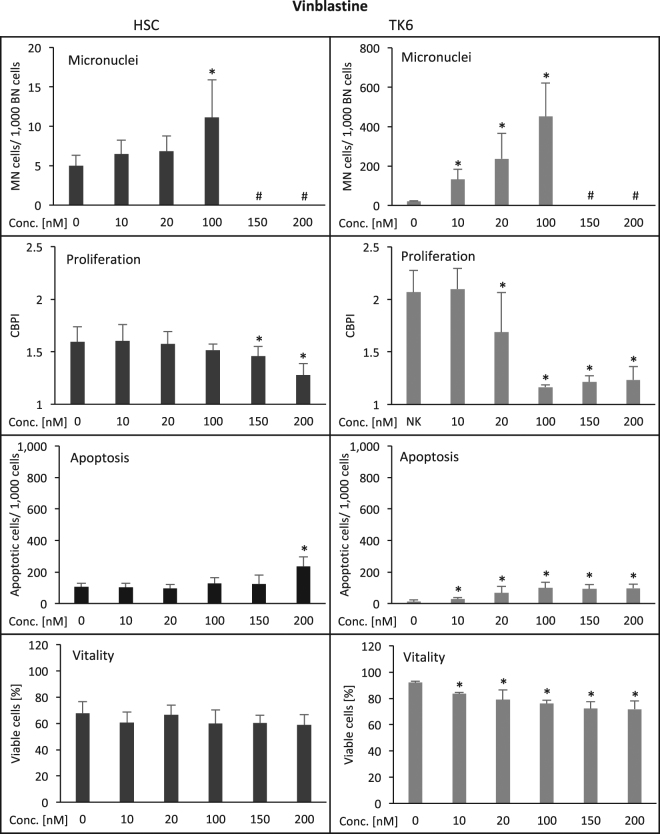
Induction of damage in human hematopoietic stem cells (HSC, left column) and human lymphoblastoid cells (TK6, right column) by vinblastine. Displayed are the frequency of micronucleated cells/1,000 binucleated cells, the reduction of cell proliferation (CBPI = cytochalasin B proliferation index), the induction of apoptosis (determined microscopically according to nuclear morphology; number/1,000 cells) and the reduction of cell viability (cell membrane integrity/enzymatic dye activation assay (%). Numbers are averages from three independent experiments with standard deviation. *p ≤ 0.05 (Mann-Whitney-U-Test). ^#^Due to toxicity, insufficient binucleated cells for micronucleus evaluation were available. Y-axes are equal for HSC and TK6 for all endpoints except for micronuclei, where scalings were chosen according to optimal visibility of effects.

The vinca alkaloid vinblastine caused elevated frequencies of micronucleated cells in HSC at 100 nM (Fig. 3, Table 1) and in TK6 cells at 10 nM. Proliferation was reduced after treatment with 150 nM vinblastine in HSC and with 20 nM in TK6 cells, while apoptosis was induced in HSC with 20 nM and in TK6 with 10 nM. Viability was not significantly affected by the tested doses in HSC and was reduced by treatment with 10 nM vinblastine in TK6 cells. Thus, HSC were 10-fold less sensitive for damage induction by vinblastine in the micronucleus test and 7.5/20-fold less regarding proliferation and apoptosis, considering only the tested doses (Table 1).

**Figure 4:**
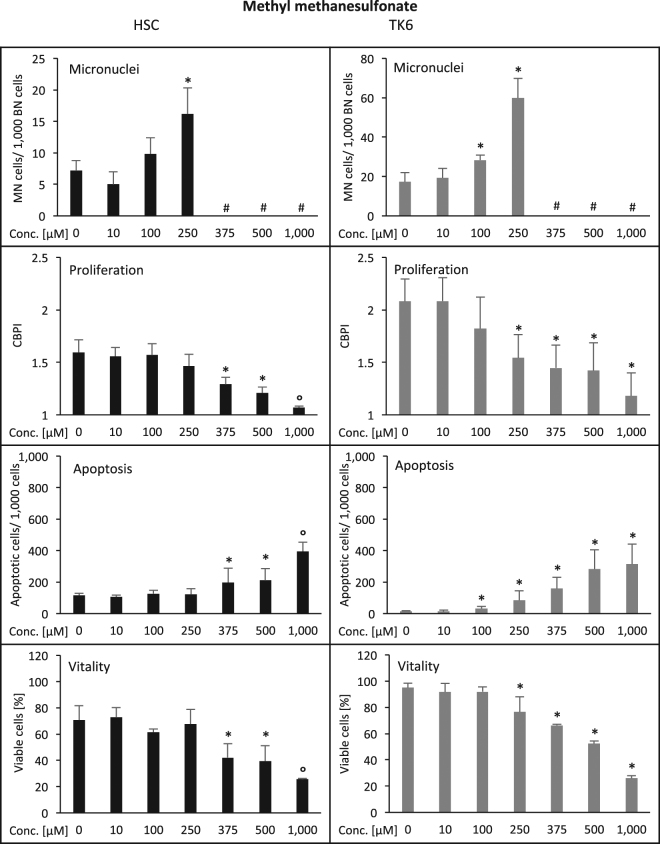
Induction of damage in human hematopoietic stem cells (HSC, left column) and human lymphoblastoid cells (TK6, right column) by methyl methanesulfonate (MMS). Displayed are the frequency of micronucleated cells/1,000 binucleated cells, the reduction of cell proliferation (CBPI = cytochalasin B proliferation index), the induction of apoptosis (determined microscopically according to nuclear morphology; number/1,000 cells) and the reduction of cell viability (cell membrane integrity/enzymatic dye activation assay (%). Numbers are averages from three independent experiments with standard deviation. *p ≤ 0.05 (Mann-Whitney-U-Test). ^°^Due to toxicity, evaluation could not be completed in all three independent repeat experiments and significance was not calculated. ^#^Due to toxicity, insufficient binucleated cells for micronucleus evaluation were available. Y-axes are equal for HSC and TK6 for all endpoints except for micronuclei, where scalings were chosen according to optimal visibility of effects.

MMS, an alkylating agent, induced micronucleus formation in HSC cells after treatment with 250 µM, while TK6 cells showed an increase after 100 µM (Fig. 4; Table 1). Proliferation, apoptosis and viability were affected in HSC with 375 µM MMS, while in TK6 cells 250 µM, 100 µM and 250 µM, respectively, were required. Again, HSC were less sensitive than TK6 cells, albeit with a smaller factor of 2.5 (micronucleated cells), 1.5 (proliferation and vitality) and 3.75 (apoptosis) (Table 1) than for the test compounds mentioned above.

**Figure 5:**
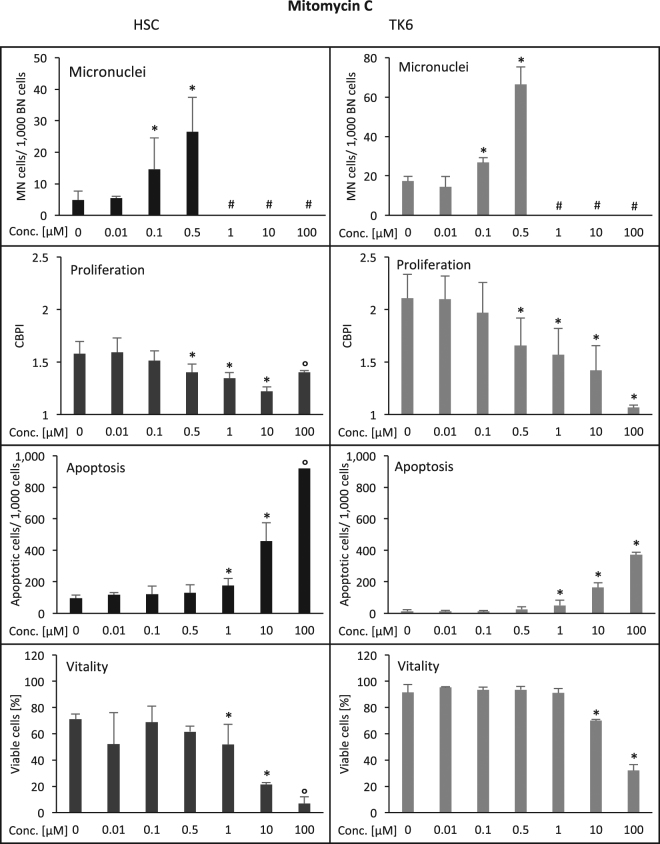
Induction of damage in human hematopoietic stem cells (HSC, left column) and human lymphoblastoid cells (TK6, right column) by mitomycin C (MMC). Displayed are the frequency of micronucleated cells/1,000 binucleated cells, the reduction of cell proliferation (CBPI = cytochalasin B proliferation index), the induction of apoptosis (determined microscopically according to nuclear morphology; number/1,000 cells) and the reduction of cell viability (cell membrane integrity/enzymatic dye activation assay (%). Numbers are averages from three independent experiments with standard deviation. *p ≤ 0.05 (Mann-Whitney-U-Test). ^°^Due to toxicity, evaluation could not be completed in all three independent repeat experiments and significance was not calculated. ^#^Due to toxicity, insufficient binucleated cells for micronucleus evaluation were available. Y-axes are equal for HSC and TK6 for all endpoints except for micronuclei, where scalings were chosen according to optimal visibility of effects.

For the effects of MMC, both cell types, HSC and TK6, showed equal sensitivity in that 0.1 µM induced micronuclei, 0.5 µM reduced proliferation and 1 µM caused elevated apoptosis (Fig. 5; Table 1). HSC cells were more sensitive for loss of viability which occurred at 1 µM while in TK6 cells 10 µM were required for a significant effect. Therefore, the factor between the effective doses was 1 for micronucleated cells, proliferation and apoptosis, and 0.1 for viability (Table 1).

## Discussion

The question of the feasibility of the micronucleus test is important with regard to the relevance of mutagenicity testing in hematopoietic cells. It was possible to follow a typical micronucleus test protocol with HSC with an incubation duration of 4 hours and an expression time of 20 hours under the influence of cytochalasin B. In order to obtain reasonable cell numbers, we pre-cultured HSC upon thawing and before experimental treatment for four days and cell number increased between 7- to 10-fold during that time. Vandevoorde *et al*.^16^ and Abernethy *et al*.^17^, other researchers who have used HSC in a micronucleus test approach so far, solved this limitation by adapting the assay to a mini-version^16^ and by not limiting the analysis to cells that had gone through mitosis^17^. Another way to overcome the limitations in cell number that is associated with the use of several types of stem cells might be immortalized pluripotent stem cells (iPSC). However, so far maintenance of (basal) genomic stability during culture has been one of the major issues and limitation for their use in research and therapeutic applications such as tissue regeneration (e.g. Rebuzzini *et al*.^18^, von Joest *et al*.^19^, Yoshihara *et al*.^20^, Wyles *et al*.^3^, Oliveira *et al*.^21^). At this stage, they can therefore not be recommended for genotoxicity testing.

To be able to rule out influences of cell density or protein content in the medium, which is an important factor in the toxic effects of substances tested *in vitro*^22–24^ we adjusted the cell density of the two investigated cell types (HSC and lymphoblastoid TK6 cells) and the protein content of the HSC medium to that of the TK6 cell culture medium during substance treatment. In the case of Vandevoorde *et al*.^16^ protein concentration was not of equal relevance for comparison of the sensitivity of different cell types, since they only used irradiation as genotoxic treatment. Abernethy *et al*.^17^ used the genotoxic chemical 1,4-benzoquinone, but did not compare cell types and therefore did not have to consider protein concentrations.

In our investigation and for the tested compounds and conditions, HSC were less sensitive and in one case (MMC) equally sensitive for genomic damage induction. For micronucleated cells and for proliferation, the order of sensitivity difference was doxorubicin >vinblastine >MMS > MMC. The only case where HSC were more sensitive than TK6 cells was their loss of viability after MMC treatment. It remains to be seen whether the results observed here can be extrapolated to the chemical classes or mechanistic classes that were represented by the selected chemicals. It also remains to be seen whether there might be a compound for which HSC are more sensitive regarding genotoxicity. However, at least the current results do not seem to indicate that genotoxicity/mutagenicity testing with the established cell line TK6 is in danger of being insufficiently sensitive regarding the true target for carcinogenesis, the stem cells. Of course, other types of stem cells, cell lines and substances would have to be investigated to be able to further support and generalize this preliminary conclusion. In support of our findings, Vandevoorde *et al*.^16^ found HSC to be less sensitive for micronucleus induction by irradiation with 2 Gy than newborn or adult T-lymphocytes. When we compared HSC with the human promyelocytic HL-60 leukemia cell line for induction of cellular and DNA-damage (comet-assay detection) after exposure to mobile phone radiation, we found no effect and therefore no difference in sensitivity between the two cell types for mobile phone radiation; the chemically induced DNA-damage (150 µM MMS; 4 h, positive control; 9.86 ± 1.84% DNA in tail for HSC; 10.85 ± 3.58% DNA in tail for HL-60) was not significantly different between the two cell types^15^. However, the damage that is observable in the comet assay is in principle repairable and does not represent a type of mutation, whereas micronucleus induction is considered a type of chromosomal mutation^25^.

The basal micronucleus frequency of cell lines can vary from around 5 to 50 micronucleated cells per 1,000 binucleated cells, and usually the more genetically stable cell lines with less than about 20/1,000 are preferred for genotoxicity testing. Here, TK6 cells exhibited 17.2 ± 4.5 micronucleated cells per 1,000 binucleated cells. HSC showed a lower frequency of 5.5 ± 2.8 micronucleated cells per 1,000 binucleated cells (average and standard deviation of 24 determinations at different times from the same original cell batch frozen as a set of vials). Vandevoorde *et al*.^16^, in their HSC (umbilical blood cord derived CD34+ HSC cells from 7 donors) found 11 micronuclei in 1,000 binucleated cells as basal frequency. This difference might be due to inter-individual variation which manifested in the investigation of Vandevoorde *et al*.^16^ but could not influence our results. They also determined the proliferation index and found an average of 1.58 ± 0.13 for the CD34+ HSC, which is almost identical to our value of 1.58 ± 0.10. Abernethy *et al*.^17^ detected 1.9 ± 0.3 micronuclei in 1,000 cells (10 donors), but because they did not limit the analysis to cells which had gone through mitosis (no addition of cytochalasin B) their micronucleus frequencies cannot be compared. With the addition of cytochalasin B, a possible difference in proliferation between cell types cannot influence the sensitivity for micronucleus induction, because analysis is normalized to actively dividing cells. However, the assay is easier to perform if proliferation is good, because a higher percentage of the treated cells can be evaluated for the presence of micronuclei.

Stem cells are thought to control their genomic integrity tightly^3,4^ although chromatin remodeling within the differentiation or proliferation process, which seems to be associated with a switch in the use of DNA damage response systems, may involve phases or situations of elevated DNA damage^26,27^. The HSC tested here are already differentiated to some degree and further differentiate during culture. The frequency of micronucleated cells which was detected as basal level may be influenced by the state of differentiation or by the cell culture conditions, which may not resemble physiological conditions within stem cell niches in the human body or the bloodstream sufficiently. Especially, the concentration of oxygen and of oxidative stress has been found to be highly relevant, because recruitment of stem cells for proliferation and differentiation is mediated at least partially by reactive oxygen species^28,29^. In mice, when hematopoietic stem cells were activated to enter the cell cycle, an increase in DNA breakage was observed and an elevated reactive oxygen species production was identified as an upstream event^27^. In fact, high ROS levels are known to cause DNA damage^30^. Therefore, maintenance of ROS levels is even more crucial for stem cells than for normal primary cells or transformed cell lines. One example how that maintenance might be compromised during *in vitro* culture is that glucose concentration in the culture medium influences cellular ROS levels due to effects on mitochondria in human mesenchymal stem cells^31^. Another example is that undifferentiated human bone marrow stromal cells required selenium supplementation to restore the antioxidative capacity and to reduce the basal micronucleus frequency during *in vitro* culture^32^. It may therefore be not surprising that iPSCs have been found to harbor upregulated antioxidant proteins^33^.

As discussed by Vandevoorde *et al*.^16^, evidence is accumulating that stem cells are equipped with very efficient DNA damage repair. In human HSC, using γ-H2Ax as marker for DNA damage, DNA repair capacity was found to be enhanced in comparison with mature lymphocytes^34^. Hyperactive CHK1 signaling occurs to control and avoid proliferation in cases where error-free DNA repair is compromised^35^. But Milyavsky *et al*.^36^ showed that the DNA damage response in HSC is affected by the degree of maturation within that still heterogeneous population. Therefore, if HSC are to be further developed for a standard micronucleus test protocol, a more in depth characterization of sensitivities of subtypes would be useful.

No direct comparison of possible genomic differences between TK6 as long existing cell line and HSC has been published, but in a whole genome sequencing approach TK6 cells have been found „very close to a standard human genome“ with some mutations that also occur as polymorphisms in the human population^37^. In DNA-damage measurements with a modified comet-assay, TK6 were found more sensitive than several other cell lines for aphidicolin, which inhibits DNA repair synthesis and leads to an accumulation of DNA incisions^5^. In addition to the demonstration of the suitability of HSC for a standard type micronucleus analysis our investigation also further supported that TK6 cells exhibit a good sensitivity for chemical mutagenesis studies, which was even superior to that of HSC with our chemicals. Altogether, it will be interesting to investigate underlying molecular causes for differences in sensitivity between HSC or stem cells in general and lymphocytes, primary cells or permanent cell lines further.

## Material and Methods

### Chemicals

Horse serum was purchased from Biochrom AG (Berlin, Germany). The protein-assay dye reagent concentrate was from Bio-Rad (Munich, Germany) and the GelGreen nucleic acid gel stain was from Biotium (Hayward, CA, USA). Hematopoietic Growth Medium (HPGM) was from Lonza (Cologne, Germany). Recombinant human fms-related tyrosine kinase 3 ligand (Flt3), purified recombinant human stem cell factor (SCF) and recombinant human thrombopoietin (TPO) were from MACS Miltenyi Biotec (Gladbach, Germany). Mitomycin C was from Medac (Hamburg, Germany) and methanol was from Carl Roth (Karlsruhe, Germany). 1,4-Diazabicyclo[2.2.2]octane (DABCO), albumin from human serum, cytochalasin B, L-glutamine solution, methyl methanesulfonate, penicillin-streptomycin (10,000 units penicillin and 10 mg streptomycin per ml), RPMI 1640 medium (HEPES modification) and sodium pyruvate solution were purchased from Sigma-Aldrich (Munich, Germany). Doxorubicin hydrochloride, mitomycin C and vinblastine sulfate were from Teva (Ulm, Germany).

### Cell culture

TK6, a human B-lymphoblastoid cell line, was received from Dr. W.J. Caspary, NIEHS, RTP, USA and was grown in RPMI 1640 medium supplemented with 10% horse serum, 1% glutamine, 1% sodium pyruvate and 0.4% penicillin/streptomycin.

The hematopoietic cord blood CD34+ stem cells (HSC) were obtained from Lonza (Cologne, Germany) and were cultured in HPGM supplemented with 50 ng/ml TPO, 50 ng/ml Flt_3_ and 25 ng/ml SCF at a starting cell density of 9,500 cells/ml. On the third day after thawing the cells were subcultured to adjust the cell density again to 9,500 cells/ml. On the fourth day after initiation of culture, the micronucleus frequency test was performed. The choice of duration of expansion time for the cells prior to the micronucleus test was based on preliminary experiments in which we had analyzed the proliferation after addition of the cytokinesis inhibitor cytochalasin B.

All cultures were kept at 37 °C in a humidified atmosphere containing 5% CO_2_.

### Micronucleus frequency test

For treatment of TK6 cells, 1 × 10^5^ cells per 1 ml medium were incubated with 10 µl of the test substance solution in a 24-well culture plate (Sarstedt, Nümbrecht, Germany). HSC were incubated at the same cell density of 2.5 × 10^4^ cells in 250 µl culture medium supplemented with 2 mg/ml HSA with addition of 10 µl test substance solution in a 96-well culture plate (Greiner Bio-One, Frickenhausen, Germany). HSA was added to adjust protein concentrations in the medium to that of the TK6 cell culture medium. No antibiotics were added during the test substance incubation. Test substances were dissolved in water or culture medium and further diluted in medium. Thus, no addition of organic solvent to the controls was required. After an incubation time of 4 h, the medium was removed and replaced by fresh antibiotic-free medium. After that, cytochalasin B (final concentration of 3 µg/ml) was added for 20 h. Cytochalasin B induces binucleated cells by inhibition of cytokinesis, as described by Fenech and Morley^38^. Micronucleus evaluation can thus be limited to those cells that have divided exactly once since the addition of the test substance. After that, the cells were brought onto glass slides by cytospin centrifugation and fixation was achieved with −20 °C methanol overnight. For staining, cells were air-dried and stained with 10 µl GelGreen solution for 7 min and covered with DABCO solution as mounting and antifade medium. Test substance concentration were chosen on the basis of preliminary experiments such that a significant induction of damage (i.e. reduction of proliferation or viability, or increase in apoptosis) was detectable at least at the highest tested dose and that the same dose range was covered for both cell types. The frequency of micronucleated cells for each treatment was analyzed by scoring 2,000 binucleated cells (1,000 from each of two parallel slides) within one experiment for the presence of micronuclei. Three independent repeat experiments were performed. All slides were evaluated coded using a fluorescence microscope with a FITC filter at a 400-fold magnification by the same observer. Sample pictures can be found in Fig. 1.

### Cell proliferation

For analysis of cell proliferation, from the slides of the micronucleus experiments, 1,000 cells were analyzed to determine the ratio of mono- (MN), bi- (BN) and multinucleated cells (MuN) per treatment. The cytochalasin B proliferation index (CBPI) was calculated using the following formula

$${\rm{CBPI}}=\frac{1\times {\rm{MN}}+2\times {\rm{BN}}+3\times {\rm{MuN}}}{{\rm{MN}}+{\rm{BN}}+{\rm{MuN}}}$$

### Apoptosis

Together with the analysis of proliferation, a visual assessment of nuclear morphology (DNA condensed into round “apoptotic bodies” in the area of the previous nucleus) was performed to quantify apoptotic cells in 1,000 cells per treatment. Sample pictures can be found in Fig. 1.

### Cell viability

At the time of cell harvest for micronucleus analysis, 35 µl of the cell suspension were mixed with 15 µl of staining solution. The staining solution contained 12 µl fluorescein diacetate (FDA; 5 mg/ml acetone) and 2 µl GelRed Nucleic Acid Gel Stain in 2 ml phosphate buffered saline. 200 cells of this mixture were analyzed at a 200-fold magnification using a FITC filter. The proportion of viable (esterase-cleaved fluorescein yielding green fluorescence) and non-viable cells (permeable membrane allowing GelRed entry, yielding red fluorescence) was evaluated. Results are shown as percentage of viable (green) cells. Three independent experiments were performed. Sample pictures can be found in Fig. 1.

### Statistics

Statistical calculations were performed using IBM SPSS Statistics 20. For evaluation of the micronucleus frequency, cytochalasin B proliferation index, apoptosis and viability the Mann-Whitney-U test was performed for each group of samples. The results were considered significant if the p value was ≤0.05.

### Data Availability

All data generated or analysed during this study are included in this published article.
