# Author Correction: Induction of micronuclei by four cytostatic compounds in human hematopoietic stem cells and human lymphoblastoid TK6 cells

**DOI:** 10.1038/s41598-018-28929-2

**Published:** 2018-07-13

**Authors:** Henning Hintzsche, Gracia Montag, Helga Stopper

**Affiliations:** 1 Institute of Pharmacology and Toxicology, University of Wuerzburg, Versbacher Str. 9, 97078 Wuerzburg, Germany; 2 Present Address: Bavarian Health and Food Safety Authority, Eggenreuther Weg 43, 91058 Erlangen, Germany

Correction to: *Scientific Reports* 10.1038/s41598-018-21680-8, published online 20 February 2018

The Acknowledgements section in this Article was omitted. The Acknowledgements section should read:

“This publication was funded by the German Research Foundation (DFG) and the University of Wuerzburg in the funding programme Open Access Publishing.”

